# Comparison of mitochondrial DNA sequences from whole blood and lymphoblastoid cell lines

**DOI:** 10.1038/s41598-022-05814-7

**Published:** 2022-02-02

**Authors:** Chunyu Liu, Jessica L. Fetterman, Xianbang Sun, Kaiyu Yan, Poching Liu, Yan Luo, Jun Ding, Jun Zhu, Daniel Levy

**Affiliations:** 1 Department of Biostatistics, School of Public Health, Boston University, Boston, MA 02118 USA; 2 School of Medicine, Boston University, Boston, MA 02118 USA; 3 DNA Sequencing and Genomics Core, NHLBI/NIH, Bethesda, MD 20892 USA; 4 Longitudinal Studies Section, Translational Gerontology Branch, National Institute on Aging, NIH, Baltimore, MD 21224 USA; 5 System Biology Center, NHLBI/NIH, Bethesda, MD 20892 USA; 6 Population Sciences Branch, NHLBI/NIH, Bethesda, MD 20892 USA; 7 Framingham Heart Study, Framingham, MA 01702 USA

**Keywords:** Genome, Systems analysis

## Abstract

Lymphoblastoid cell lines (LCLs) provide an unlimited source of genomic DNA for genetic studies. Here, we compared mtDNA sequence variants, heteroplasmic or homplasmic, between LCL (sequenced by mitoRCA-seq method) and whole blood samples (sequenced through whole genome sequencing approach) of the same 130 participants in the Framingham Heart Study. We applied harmonization of sequence coverages and consistent quality control to mtDNA sequences. We identified 866 variation sites in the 130 LCL samples and 666 sites in the 130 blood samples. More than 94% of the identified homoplasmies were present in both LCL and blood samples while more than 70% of heteroplasmic sites were uniquely present either in LCL or in blood samples. The LCL and whole blood samples carried a similar number of homoplasmic variants (*p* = 0.45) per sample while the LCL carried a greater number of heteroplasmic variants than whole blood per sample (*p* < 2.2e−16). Furthermore, the LCL samples tended to accumulate low level heteroplasmies (heteroplasmy level in 3–25%) than their paired blood samples (*p* = 0.001). These results suggest that cautions should be taken in the interpretation and comparison of findings when different tissues/cell types or different sequencing technologies are applied to obtain mtDNA sequences.

## Introduction

Whole blood and cell lines are two common sources of DNA for genetic studies. In contrast to the a limited supply of DNA from whole blood samples, an Epstein–Barr Virus (EBV) transformed lymphoblastoid cell line (LCL) provides an unlimited source of genomic DNA^1^. Concerns regarding the use of LCL-derived DNA for genetic studies have been raised because EBV transformation may generate a small number of de novo mutations^2–4^ and introduce locus-specific biases^5^. With regard to nuclear DNA variants assessed, several studies have found that genotypic discordance is negligible in DNA obtained from LCL samples with a low number of frozen/thawed processes (i.e., low-passage) compared to whole blood-derived DNA^2–4,6,7^. Humans have two sets of genomes, the nuclear DNA and the mitochondrial genome (mtDNA); the latter plays a critical role in energy production through oxidative phosphorylation (OXPHOS). The mtDNA is a maternally inherited, small genome (16,569 base pairs) that encodes 37 genes including 13 genes for OXPHOS proteins, 2 for rRNAs, and 22 for tRNAs. Each cell contains many copies of mtDNA molecules, and therefore, mutations can either affect all mtDNA molecules (termed homoplasmy) or a proportion of mtDNA molecules (termed heteroplasmy)^8^. To date, it remains unclear if LCL samples is suitable to be used as an unlimited source (i.e., the replacement for whole blood) to study mtDNA sequence variations.

A previous study^9^ was aimed to extract and annotate human mtDNA from the whole exome sequencing (WES) data in the 1000 Genomes Project^10^. The mean coverage of the WES sequences was about fourfold per base for the nuclear genome and 25- to 409-fold per base for mtDNA^9^. Within all study samples, a subset of 60 LCL samples and 60 independent blood samples were identified, and mtDNA sequence variations were compared between the two DNA sources. About a quarter of the identified sequence variations in mtDNA were present in both the 60 LCL and 60 blood samples. Most (80%) of the quarter of the variations that were present in both types of samples were homoplasmy. In addition, this study found that LCLs trended to accumulate low-level heteroplasmies (heteroplasmy level < 10%) than whole blood samples, while the two DNA sources had a comparable average number of heteroplasmic sites per sample (7.42 for LCLs, 7.33 for blood samples)^9^. Of note, this previous study compared the mtDNA sequence variations in independent LCL and blood samples with WES of low depth coverage. mtDNA heteroplasmy is optionally studied with deep sequencing (> 1,000-fold). Therefore, the comparability of mtDNA sequence variations, particularly heteroplasmic variations, between the LCL samples and blood samples of the same individuals with deep sequencing remained to be studied.

Recently we received mtDNA deep sequencing from LCL samples from 365 Framingham Heart Study (FHS) participants with the mitoRCA-seq method, a targeted sequencing technology^11^. We also received whole genome deep sequencing (WGS) (~ 30-fold coverage per base in nuclear DNA) from whole blood from 4,100 FHS participants through the Trans-Omics for Precision Medicine (TOPMed) program supported by the National Heart, Lung, and Blood Institute’s (NHLBI)^12^. We isolated, among the above samples, LCL and blood, mtDNA sequencing data from 130 participants (45% women). Therefore, this present study was aimed to investigate the comparability of mtDNA sequencing variations (homoplasmy and heteroplasmy) between the paired LCL samples and whole blood samples of the same 130 FHS participants. Because two different sequencing technologies were applied to obtain mtDNA sequences, we performed harmonization to obtain comparable sequences coverage depths to mtDNA sequences from the two technologies. We also performed comprehensive and consistent quality control procedures to identify mtDNA sequence variations from the two DNA sources.

## Results

**Figure 1:**
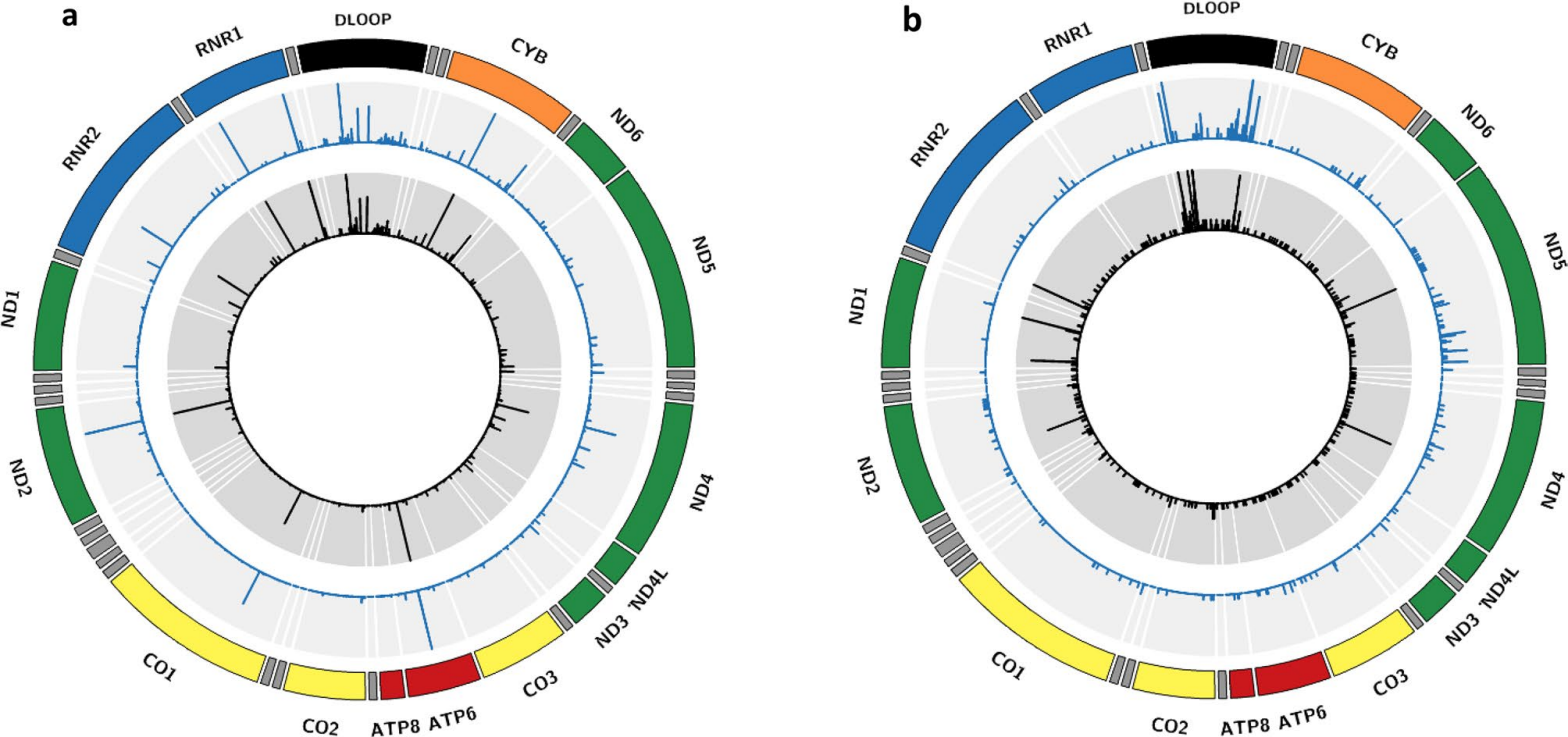
Comparison of homoplasmy (**a**) and heteroplasmy (**b**) between the 130 paired LCL (inner circle with black strokes) and whole blood (middle circle with blue strokes) samples from 130 Framingham Heart Study participants. The height of strokes represents frequency of a variation in mtDNA in the 130 participants. The major genes encoded by mtDNA is plotted in outer circle. LCL, lymphoblastoid cell line.

After harmonization procedures (see Methods), we obtained comparable sequence coverage depth (mean coverage 2,000-fold) between the 130 LCL and blood samples (Supplementary Fig. 1). With an alternative allele fraction (AAF) 3–97% threshold (see Methods, Supplementary Table 1), we identified 866 sites being homoplasmic and/or heteroplasmic in the 130 LCL samples; we also identified 666 sites in the 130 blood samples (Fig. 1). Below we present detailed comparisons for the homoplasmies and heteroplasmies between the paired LCL and blood samples from the same 130 participants.

### Comparison of homoplasmy between LCL- and whole blood-derived DNA samples

**Figure 2:**
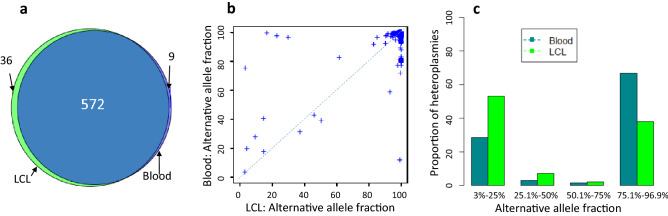
Comparison of homoplasmic sites between the paired LCL and whole blood samples from 130 Framingham Heart Study participants. (**a**) Venn diagram of the observed homoplasmic sites in LCL and whole blood samples. (**b**) Comparison of the AAFs (%) of the 572 homoplasmic sites that are present in both LCL and whole blood samples. (**c**) Comparison of the AAFs of 119 (of 581) and 66 (of 608) sites being heteroplasmic between LCL and whole blood samples (**c**). AAF, alternative allele fractions. LCL, lymphoblastoid cell line.

A site was defined as homoplasmic in a participant if AAF > 97%. We observed 608 sites being homoplasmic in the 130 LCL samples after harmonization, while we observed a slightly lower number of sites (n = 581) being homoplasmic in the 130 blood samples (608/16,569 versus 581/16,569, *p* = 0.44, where 16,569 is the total number of base pairs in mtDNA). The median number of homoplasmic sites was 27 (interquartile range = 12, 32) per LCL sample and was 25 (interquartile range = 12, 33) per whole blood sample (Supplementary Fig. 2). Therefore, the LCL or blood samples harbored a similar number of homoplasmic sites per sample (27 versus 25, Wilcoxon signed rank test *p* = 0.45) (Supplementary Fig. 2). Of all homoplasmies observed, 572 sites (94% in LCL samples or 98% in whole blood samples) were present in both LCL and blood samples, i.e., they were at the same base pair locations and had the same alternative alleles in both LCL and blood samples) (Fig. 2a). As expected, the haplogroups identified for the 130 FHS participants were identical using the homoplasmies in the 130 LCL and 130 whole blood samples (Supplementary Table 2). Most homoplasmies identified (~ 88%) were observed in fewer than 5% participants in both LCL and whole blood samples (Supplementary Table 3). The observed frequencies of the identified homoplasmies in the 130 LCL or whole blood samples were consistent with those in 51,836 human mitochondrial full sequences (Pearson correlation *r* = 0.95) from in GenBank through MITOMAP^13^ (Supplementary Table 3).

We investigated the AAF distributions for the identified 608 sites in the 130 LCL samples and 581 in the 130 whole blood samples (Fig. 2b). Among the 608 sites being homoplasmic in LCL samples, 66 (10.8%) were heteroplasmic (i.e., AAFs < 97%) in 41 of the 130 LCL samples. Similarly, among the 581 sites being homoplasmy in blood samples, 117 (20.4%) sites were heteroplasmic in 62 of the 130 blood samples (20.1% versus 10.7%; two proportions z-test *p* = 5e−6 (Supplementary Tables 4 and 5). We further compared the heteroplasmy levels (i.e., AAF distributions) of these 66 (41 LCL samples) and 117 (62 whole blood samples) sites at four AAF intervals, including (1) 3–25% (low level), (2) 25.1–50% (low-to-middle level), (3) 50.1–75% (middle-to-high level), and (4) 75.1–96.9% (high level). In whole blood, 28.7%, 2.9%, 1.4%, and 67.0% of the heteroplasmies at the 117 sites were observed at the low level, low-to-middle level, middle-to-high level, and high level interval, respectively. In LCL samples, 53.1%, 7.1%, 2.0% and 37.8% of heteroplasmies at these 66 sites were observed at 3–25%, 25.1–50%, 50.1–75%, and 75.1–96.9% interval, respectively. A greater proportion of the 117 sites were high-level heteroplasmies than that of the 66 sites (67.0% versus 37.8%, two proportions z-test *p* = 2.8e−5) (Fig. 2). The finding that a higher number of mtDNA sites in whole blood than LCL samples were both heteroplasmy and homoplasmy and two third of such sites were at a high heteroplasmy level may reflect the fact that a mixture of mtDNA molecules exist in whole blood of various white blood cells and thus, the alternative allele fractions of certain homoplasmies may slightly vary across the white blood cells. We further compared the locations of these 66 (in LCL) and 117 (whole blood) heteroplasmic sites, and found that these sites were distributed similarly in their proportions across the D-loop region, coding genes, rRNA genes or tRNA genes (two proportions z-test *p* > 0.05) (Supplementary Table 6a). Among the coding heteroplasmic variants (66 in LCL and 117 in whole blood), nonsynonymous (3 versus 15, Fisher’s exact test *p* = 0.16) or deleterious variants, defined by the combined annotation-dependent depletion (CADD) PHRED-like score^14^ ≥ 15 (1 versus 4, Fisher’s exact test *p* = 1) were not significantly different in their proportions in LCL and whole blood samples (Supplementary Table 6b).

### Comparison of heteroplasmies between LCL- and whole blood-derived DNA samples

**Figure 3:**
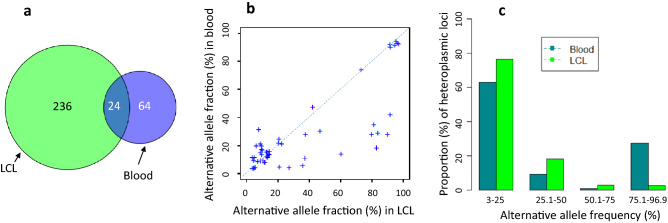
Comparison of heteroplasmic sites between 130 paired LCL and whole blood samples. (**a**) Venn diagram of the observed homoplasmic sites in LCL and whole blood samples. (**b**) Comparison of the AAFs (%) of 24 heteroplasmic sites that are present in both LCL and whole blood samples. (**c**) Comparison of the AAFs of 258 and 85 heteroplasmic sites between LCL and whole blood samples. AAFs, alternative allele fractions. LCL, lymphoblastoid cell line.

In this session, we compared heteroplasmic sites only if their AAFs were present between 3 and 97%, that is, none of these sites were homoplasmic in any of the paired samples. We observed 258 base pair positions (i.e. sites) in LCL samples after harmonization (Supplementary Table 7). Compared to the 130 LCL samples, the 130 blood samples had a much lower number of heteroplasmic sites (*n* = 85; 85/16,569 versus 258/16,569, two proportions z-test *p* < 2.2e−16) (Supplementary Table 8). At an individual level, a LCL sample carried a much higher number of heteroplasmic sites (median = 7 with interquartile range = 5, 8) than a blood sample (median = 1 with interquartile range 0, 3; Wilcoxon signed rank test *p* < 2.2e−16) (Supplementary Fig. 3). Among all heteroplasmic sites, 24 were present in both LCL and blood samples (Fig. 3a), and about 60% of these 24 sites displayed consistent AAFs between the paired samples (Fig. 3b). Among the 258 (in LDL) and 85 (in whole blood) heteroplasmic sites, those locating in the D-loop region, coding genes, rRNA genes or tRNA genes were not significantly different in their proportions (two proportions z-test *p* > 0.05) (Supplementary Table 6a). However, a higher proportions of coding variants were nonsynonymous (115 versus 29, two proportions z-test *p* = 0.00044) or deleterious (CADD PHRED-like score ≥ 15) (77 versus 20, two proportions z-test *p* = 0.048) in LCL than in whole blood samples (Supplementary Tables 7 & 8). We further compared the two types of heteroplasmic sites (i.e., the sites with AAFs only in the 3–97% range in this section and the sites that being both heteroplasmic and homoplasmic in the proceeding section) in LCL samples, and we observed that the former were significantly enriched with nonsynonymous (Fisher’s exact test *p* = 1.5e−9) and deleterious (Fisher’s exact test *p* = 4.0e−6) variants than the latter (Supplementary Table 6b). Similarly, the former were significantly enriched with nonsynonymous (two proportions z-test *p* = 0.028) and deleterious (Fisher’s exact test *p* = 0.0004) variants than the latter in whole blood samples.

In both the 130 LCL and blood samples, most heteroplasmies were present in only one sample. More specifically, 212 (82.2%) heteroplasmic sites were present in only one LCL sample, and 76 (89.4%) sites were present in only one blood sample (82.2% versus 89.4%, two proportions z-test *p* = 0.43). The rest of heteroplasmic sites were present in 2 to 14 samples in both LCL and blood sample, except that eight sites were found in more than 14 LCL samples, and among which one of them (position 3106 in *RNR2*) was present in 126 LCL samples. It appeared that most of these heteroplasmy sites were also rarely (< 0.1%) as variation sites across 51,836 human mitochondrial full sequences in GenBank through MITOMAP^13^ (Supplementary Tables 7 and 8).

We compared the heteroplasmy levels of the observed heteroplasmies between the paired samples. The median value of heteroplasmy levels was lower in LCL samples (median AAF = 8%) than that in whole blood samples (median AAF = 15%; 8% versus 15%, Wilcoxon signed rank test *p* = 0.0003). We further compared the heteroplasmy levels across the four AAF intervals. In LCL samples, 76.4% heteroplasmies were at the low level (AAF 3–25%), 18.2% at low-to-middle (25.1–50%), 2.8% at middle-to-high (50.1–75%), and 2.6% at high level (AAF 75.1–96.9%). In blood samples, 62.9%, 9.1%. 0.8%, and 27.2% heteroplasmies were observed at the low level, low-to-middle level, middle-to-high level, and high level, respectively (Fig. 3c). These observations showed that the LCL samples had a higher proportion of heteroplasmies at low level (76.4% versus 62.9%, two proportions z-test *p* = 0.001) and low-to-middle level (18.2% versus 9.1%, two proportions z-test *p* = 0.015) than blood samples. A greater number of heteroplasmic sites were found in LCL and a higher proportion of heteroplasmies at low level may result from a selection of mtDNA populations followed by enrichment during the subsequent processes after EBV-transformation. In contrast, blood samples had a much higher proportion of heteroplasmies at high-level than LCL samples (27.2% versus 2.6%, two proportions z-test *p* < 2.2e−16) (Fig. 3c). Again, this may reflect a mixture of mtDNA molecules in whole blood.

## Discussion

We compared the mtDNA homoplasmic and heteroplasmic sites between the 130 paired LCL and blood samples of the same participants in the FHS. Although most (> 94%) of the identified homoplasmies were present in both the LCL and blood samples, most heteroplasmies were uniquely present in either LCL or blood samples. In addition, we observed a much higher number of heteroplasmic sites per LCL sample than per blood sample in this study. Furthermore, we found that the LCL samples tended to accumulate low level heteroplasmies than their paired blood samples, which was consistent with an observation from a previous study^9^. Although these heteroplasmic sites were distributed similarly in their proportions across mtDNA genomic regions between LCL and whole blood samples, higher proportions of coding heteroplasmic sites were found to be nonsynonymous or deleterious in LCL than in whole blood samples. This difference may result in changes in their energy production processes in LCL compared to whole blood samples.

Of note, two sequencing technologies were used to obtain mtDNA sequencing data from LCL samples and blood samples. The LCL samples were sequenced by a targeted sequencing technology, mitoRCA-seq approach^15^ while blood samples were sequenced by WGS method^12^. Due to the use of two different technologies, a much higher coverage depth was obtained for LCL samples than their paired blood samples (see Methods). To minimize the effects of different coverage depths on the identification of heteroplasmies, we applied the harmonization of sequencing reads to obtain comparable coverage depths between the paired samples. However, the use of different sequencing technologies may partly contribute to the observed differences between paired samples. Nevertheless, other observed differences between the LCL and blood samples are not readily explained by different sequencing technologies. First, a higher number of homoplasmic sites displayed a wide range of AAFs across the 130 blood samples than the 130 paired LCL samples. This may reflect the fact that whole blood contains a variety of white blood cells that have a mixture of mtDNA populations as opposed to LCLs that consists of mainly B cells after transformation by EBV^1,16^. It is unclear if the differences in mtDNA populations occur very early during embryonic development, prior to differentiation events, or during aging. Second, the LCL samples harbored a significantly higher proportion of low (AAF 3–25%) or low-to-middle level (AAF 25.1–50%) heteroplasmic sites than blood-derived DNA samples. These low-level variants in mtDNA may be undetectable in whole blood with different blood cells. However, these low-level variants may undergo clonal expansion and bottleneck phenomenon of the mtDNA^17,18^ after the EBV-transformation, and therefore, they reached a detectable level in the LCL samples that mainly consist of B cells^1,16^. Third, the mitochondrial adaption to the new energy requirement in EBV-transformed LCLs may further select mtDNA populations for enrichment during the subsequent processes after the EBV-transformation.

The findings in this study have three important inferences. First, the findings in this study suggest that an extreme caution must be taken in the interpretation and comparison of study results when different tissue or cell types (i.e., LCL versus whole blood) are used in the analysis of mtDNA heteroplasmies, despite that immortal EBV-transformed LCL technique has been established as an infinite source of DNA. Second, extreme cautions must be taken to interpret results in using induced Pluripotent Stem Cells (iPS) to study heteroplasmic functions. A previous study observed varying burdens of heteroplasmy in iPS cells generated by reprogramming somatic cells^19^. Third, a caution is also needed to compare heteroplasmies in samples that are sequenced by different sequencing technologies. Due to the rapid evolvement of technologies, it is common that mtDNA is sequenced by different sequencing technologies across cohorts in a large collaboration. Harmonization of sequencing data from different sequencing technologies and consistent quality control procedures are critical to minimize the bias that may result from different sequencing technologies.

## Methods

### Study population

Beginning in 1948, the FHS has enrolled three generations of participants from the town of Framingham in Massachusetts^20–22^. This study included participants from the Offspring cohort^21^ and the Third Generation cohort^22^ who have been examined every four to eight years. Recently we received mtDNA sequences of the LCL-derived mtDNA samples from the 365 unrelated FHS participants^11^. We also received WGS data in whole blood from 4,100 participants through the NHLBI’s TOPMed program. We identified 130 participants (45% women) who received mtDNA sequencing with both LCL-derived and whole blood-derived DNA samples. The 130 participants were middle-aged (mean age 43–47 years) at the EBV transformation of the LCL and for the whole blood draw. All study participants provided written informed consent for using their DNA for genetic research and cell line creation. All experimental protocols were approved by the Boston Medical Center and Boston University Medical Campus Institutional Review Board (IRB). All experiments and analyses were performed in accordance with relevant guidelines and regulations.

### DNA extraction

The genomic DNA was extracted from LCLs by the following procedures: 100 ml of saturated cell suspension was centrifuged at 1500 rpm for 10 min to form a cell pellet. The pellet was washed twice using 0.85% NaCl solution. The pellet was then re-suspended in 4.3 ml of cell lysis buffer solution (pH 7.2) that contained 0.01 M KCl, 0.4 M NaCl, 0.01 M MgCl_2_, 0.1 M Tris, and 0.002 M EDTA. Next, 135 µl of 20% SDS solution and 10 µl of RNase were added to the suspension. The mixture was incubated at 37 °C for 1 h following which, 35 µl of proteinase K (41.7 mg/ml) was added. The mixture was incubated at 56 °C for an additional 2 h after which 1.6 ml of 6 M NaCl was added. The solution was mixed well and centrifuged at 3350 rpm for 15 min. The supernatant was collected and mixed with an equal volume of isopropanol to precipitate the DNA. The genomic DNA pellet was collected following centrifugation at 2500 rpms for 3 min after which, the pellet was washed using 70% ethanol. The genomic DNA was stored in 1 × TE solution.

The extraction of genomic DNA from buffy coat/whole blood used the following procedure. The red blood cells were lysed by adding 50 mL of lysis buffer (tromethamine, sucrose, MgCl_2_, triton) to 5 mL whole blood. The white blood cell pellet was collected following centrifugation. To lyse the white blood cells, 2 ml white blood cell lysis solution (tromethamine, EDTA and NaCl) was added to the pellet, followed by the addition of 0.5 ml 5 M sodium perchlorate. The mixture was incubated at 65 °C for 1 h. Two mL of phenol/chloroform was added to the mixture after incubation. The mixture was centrifuged for 30 min at 2,800 rpm to obtain the DNA pellet. The pellet was washed with 70% ethanol and re-suspended in 1 X TE.

### mtDNA sequencing and quality control

#### Sequencing in LCL-derived DNA samples

A targeted approach was used for sequencing the LCL-derived DNA samples, which was previously described^11^. Briefly, the National Heart, Lung, and Blood Institute’s (NHLBI’s) DNA Sequencing and Genomics Core Facility performed mtDNA sequencing using the mitoRCA-seq approach^15^. This approach employed a rolling circle amplification (RCA) method to enrich full length mtDNA molecules from genomic DNA for subsequent library construction. The enrichment of full length mtDNA with a high-fidelity enzyme, Phi29 DNA polymerase, to minimize contamination from the nuclear-encoded mtDNA sequences^15^. The Illumina HiSeq-2000 platform was used for the sequencing of mtDNA. The raw reads were aligned to the human LCL- and whole-blood-derived DNA. The raw sequence reads were aligned to the human revised Cambridge Reference Sequence (rCRS)^23,24^ by Burrows-Wheeler Aligner (BWA) using default parameters^11,25^. We only used reads with mapping quality score ≥ 30 to minimize potential sequencing errors^11,26^. We then selected the reads with the highest average quality score (defined as best-unique read) for raw reads sharing the same sequence. Per base median coverage was 4157-fold (inter quartile range 2846 to 5444) from the 130 LCL-derived DNA samples.

#### Sequencing in whole blood-derived DNA sample

Whole genome sequencing of genomic DNA from whole blood was conducted in ~ 4,100 FHS participants at the Broad Institute of MIT and Harvard through NHLBI’s TOPMed project. DNA fragmentation and library construction followed standard procedures. Sequencing was performed using Hi Seq X with sequencing software HiSeq Control Software (HCS) version 3.3.76, then analyzed using RTA2 (Real Time Analysis). The DNA sequence was aligned to a human genome (hg) 19 decoy reference: picard, GATK (3.1–144-g00f68a3) and BwaMem (0.7.7-r441). A sample’s sequence was considered complete when the mean coverage of nDNA was ≥ 30x. The sample coverage and two quality control metrics (Fingerprint LOD score and proportion of contamination) were reviewed (https://www.nhlbiwgs.org/topmed-whole-genome-sequencing-methods-freeze-8). The average coverage was ~ 30-fold per base in nuclear DNA across TOPMed samples. For the 130 whole blood-derived DNA samples, per base median coverage was 2177-fold (inter quartile range 1866 to 2578) for mtDNA sequences.

#### mtDNA variation identification

We applied MToolBox software package^27^ to the sequence reads (bam data) of the 130 LCL and whole blood samples using the mtDNA reference sequence rCRS^24^. MToolBox consequently remapped reads onto the reference nuclear genome (GRCh37/hg19) to remove nuclear mitochondrial DNA segments (NumtS). The resulting aligned reads is used to reconstruct a complete mitochondrial genome by the *assembleMTgenome.py* script and integrating the *mtVariantCaller.py* module for nucleotide mismatches and indels detection^27^. We also obtained for each site the sequence depth, alternative alleles and their frequencies (i.e., AAF) in LCL and blood samples using the MToolBox pipeline. In addition, we also obtained the haplogroups using the MToolBox pipeline. MToolBox uses Phylotree resource^28^ in predicting mtDNA haplogroups.

#### Quality control

We performed extensive quality control procedures to the sequencing data obtained from both DNA sources. In addition to a few procedures that were uniquely applied in either LCL-derived^11^ or whole blood-derived DNA samples, most procedure were consistently applied for sequences of both DNA sources. First, we excluded the sites if the coverage was less than 250 mapped reads. Second, we compared homoplasmic sites identified in sequencing data to those identified in two of earlier genotyping arrays. We previously had genotyping data for these 130 participants using two platforms: the Illumina HumanExome BeadChip (including 172 mtDNA sites)^29^ and a customized array (including 40 homoplasmic variants)^30^. A site with low (e.g., < 25%) AAF is most likely to be identified as homoplasmy of the reference allele, and a site with high (e.g., > 75%) AAF is most likely to be identified as a homoplasmy of the alternative allele in genotyping arrays. Therefore, we used a relaxed threshold to identify variation sites only for QC purposes. We compared sequencing-derived homoplasmic sites based on AAF ≥ 75% to those identified using two genotyping arrays in the same individuals. Samples showing one or more different sites were flagged. Third, we counted the number of mtDNA sites with 25% < AAF < 75% in sequencing data. Empirically, samples showing five or more such sites were flagged for further investigations. For the WGS data through TOPMed, we defined maternal lineages. Because homoplasmy is inherited through mothers, we compared homoplasmic sites in the participants of the same maternal lineages. Samples showing one or more inconsistent homoplasmic sites were flagged. We found that these multiple QC procedures consistently identified the same samples with issues. These 130 samples remained after comprehensive QC procedures.

### Harmonization of sequencing coverage in mtDNA between LCL- and whole blood-derived DNA samples

The median coverage was 4,157 in LCL-derived DNA sequences and 2,177 in whole blood-derived DNA sequences (Supplementary Fig. 1). Largely different sequence coverage in two DNA sources may contribute to the observed difference in the number of heteroplasmic sites between two DNA sources, and, therefore, we performed harmonization of sequence coverage between the paired samples with two DNA sources. We generated ten bundles of reads for LCL-derived DNA samples with comparable coverage in whole blood-derived DNA samples (Supplementary Fig. 1). We then applied the identification of homoplasmy and heteroplasmy in each sampling bundle of LCL-derived DNA sequences (see below). A locus was confirmed if it was identified in all of the ten resampling bundles.

### Identification of homoplasmy and heteroplasmy

#### Selection of threshold

In TOPMed, three FHS individuals in a parent–child trio (i.e., mother, father and a child) were sequenced simultaneously at four sequencing centers through NHLBI’s TOPMed program. Although these four centers used the same sequencing technology, minor fluctuations in sequencing reads existed across the sequencing centers. We applied four AAF thresholds (t_1_ and t_2_), 1% and 99%, 2% and 98%, 3% and 97%, and 4% and 96%, to identify the appropriate cutoffs for identification of homoplasmy and heteroplasmy in the repeated samples. A site was defined as a heteroplasmy if its AAF was between t_1_ and t_2_ (i.e., t_1_ < AAF < t_2_). A site was considered a homoplasmy of an alternative allele if AAF ≥ t_2_. We found that 3% and 97% of thresholds yielded consistent number of homoplasmic sites and heteroplasmic sites in the trio samples (Supplementary Table 1).

#### Comparison of mtDNA sequence variations

We applied the 3–97% threshold to mtDNA sequences to identify homoplasmy and heteroplasmy between two DNA sources. That is, a site was identified as homoplasmy if AAF > 97% and a site was deemed as heteroplasmy if AAF was between 3 and 97%. We excluded any sites with coverage < 250-fold, and we also excluded the following artifact prone sites: 301, 302, 310, 316, 3107, 16,182, according to the guidance from the mtDNA calling pipeline by Broad Institute (gnomAD v3.1)^31^. We compared the distribution of homoplasmic and heteroplasmic sites between the paired samples. We also compared the distribution of AAFs for identified mtDNA variation sites between paired samples. We used MToolBox^27^ to identify mtDNA sequence variations from whole blood-derived DNA samples. The identification of mtDNA sequence variations in LCL-derived DNA samples was previously described^11^. All statistical analyses for comparisons used R language^32^.

### Annotation of mtDNA sequence variations

MitoMap^13^, a human mitochondrial genome database, collects information regarding mtDNA sites, regulatory elements, and previously associated phenotypes for all identify mtDNA variants. Many functions in MitoMap was adopted by MToolBox^27^ for the annotation of the mtDNA variants. Both MitoMap and MToolBox were used to annotate the identified mtDNA variants in this study. For mtDNA sequence variations within peptide-encoding genes that were non-synonymous, the predicted functional effects were collected from MitImpact, which is a database that has compiled functional predictions across 14 bioinformatics platforms and five meta-predictors^26^.

## Supplementary Information

Supplementary Information 1.

Supplementary Information 2.
