# Supplementary material for: Comparison of mitochondrial DNA sequences from whole blood and lymphoblastoid cell lines
Source: Sci Rep. 2022 Feb 2;12:1801. doi: 10.1038/s41598-022-05814-7 (PMC8810874; doi:10.1038/s41598-022-05814-7)
Supplement: Supplementary file 1 — Supplementary Information 1. [file 41598_2022_5814_MOESM1_ESM.pdf]

Supplementary Materials

Comparison of mitochondrial DNA sequences from whole blood and lymphoblastoid cell lines

Running title: mtDNA from whole blood and lymphoblastoid cell lines

Chunyu Liu[1,*], Jessica L. Fetterman[2], Xianbang Sun[1], Kaiyu Yan[1], Poching Liu[3], Yan Luo[3], Jun Ding[4], Jun Zhu[5], and Daniel Levy[6,7,*]

[1]Department of Biostatistics, School of Public Health, Boston University, Boston, MA 02118; [2]School of Medicine, Boston University, Boston, MA 02118; [3]DNA Sequencing and Genomics Core, NHLBI/NIH, Bethesda, MD 20892; [4]Laboratory of Genetics and Genomics, NIA/NIH, Baltimore, MD 21224; [5]System Biology Center, NHLBI/NIH, Bethesda, MD 20892; [6]Population Sciences Branch, NHLBI/NIH, Bethesda, MD 20892; [7]Framingham Heart Study, Framingham, MA 01702;

[*]Correspondence:

liuc@bu.edu (C.L.); phone: 617-358-1841

levyd@nhlbi.nih.gov (D.L.); phone: 508-935-3442

Supplementary Table 1. Framingham Heart Study repeated samples by four sequencing centers in TOPMed

TOPMed ID		Mean coverage	Sequencing center	1% and 99%		2% and 98%		3% and 97%		4% and 96%	
				Het	Hom	Het	Hom	Het	Hom	Het	Hom
IND 1	NWD321439	2665	Broad	6	16	4	16	2	16	2	16
IND 1	NWD465832	2057	Illumina	9	16	4	16	2	16	2	16
IND 1	NWD433184	1675	NYGC	57	11	6	15	2	16	2	16
IND 1	NWD481739	1592	UW	13	16	3	16	2	16	2	16
IND 2	NWD813793	1846	Broad	9	23	4	25	3	25	3	25
IND 2	NWD507659	2333	Illumina	9	24	4	24	3	24	3	24
IND 2	NWD143123	1848	NYGC	10	25	4	25	3	25	3	25
IND 2	NWD284313	1844	UW	11	25	5	25	3	25	3	25
IND 3	NWD985536	1458	Broad	13	12	3	16	3	16	2	16
IND 3	NWD820361	2146	Illumina	16	14	5	16	3	16	3	16
IND 3	NWD808124	1694	NYGC	16	12	3	16	3	16	3	16
IND 3	NWD641266	1937	UW	17	14	3	16	3	16	3	16

In TOPMed, three FHS individuals in a parent-child trio (i.e., mother, father and a child) were sequenced simultaneously at four sequencing centers with the same sequencing technology. Minor fluctuations in sequencing reads existed across the sequencing centers. We applied four AAF thresholds (t1 and t2), 1% and 99%, 2% and 98%, 3% and 97%, and 4% and 96%, to identify the appropriate cutoffs for identification of homoplasmy and heteroplasmy in the repeated samples. A site was defined as a heteroplasmy if its AAF was between t_1 and t_2 (i.e., t_1<AAF< t_2). A site was considered a homoplasmy of an alternative allele if AAF≥t_2. We found that 3% and 97% of thresholds yielded consistent number of homoplasmic sites and heteroplasmic sites in the trio samples.

Supplementary Table 2. Haplogroup distribution in the 130 FHS participants

Haplogroup	Frequency	Haplogroup	Frequency	Haplogroup	Frequency	Haplogroup	Frequency
A2aj	1	H2c	1	J1c2e	1	T2b2b	1
H	5	H3+152	1	J1c3a2	1	T2b3+151	1
H+195	1	H3+16311	1	J1c5	1	T2b4	1
H1	1	H41a	1	J1c5b	1	U1b1	1
H1+152	1	H4a1a4b	1	J1c7	1	U2d	1
H1+16189	1	H5	1	J1d1a1	1	U2e1a1	1
H101	1	H5a1	2	J2a1	1	U3a	1
H10e	1	H5a1j	1	K1a	1	U3a1	1
H10f	1	H6a1b2	1	K1a+195	1	U4a1	1
H11a5	1	H6a1b3	1	K1a1b1a	1	U5a1a1	1
H13a1a2	1	H73	1	K1a4a1a2a	1	U5a1b1	1
H13a1a6	1	H7c1	1	K1c2	2	U5a1b1c1	1
H13b2	1	H7d	1	K2a10	1	U5a1g	1
H14	1	H86	2	K2a9	1	U5a2a1d	1
H18	1	HV	1	N1b1a	1	U5b1b1+@16192	1
H1a	2	HV+16311	1	N1b1b1	1	U5b2a6	1
H1a1	1	HV0f	1	R	1	U5b2b1a1	1
H1au1b	1	HV1c	1	R0a1a	1	U5b2c2b	1
H1ay	1	HV4a1a4	1	R0a2f	1	U5b3a2	1
H1ba	2	I1a1b	1	R0a3a	1	U8a1a	1
H1bb	1	I2	1	T	1	V3a1	1
H1c3b	1	I2a2	1	T1a1c	1	W5a1	1
H1e1a	1	I3	1	T1a1i	1	W6	1
H1e2	1	I3d1	1	T2	1	W6a	1
H1i	1	I6b	1	T2a1a	2	W6c	1
H1r	1	J1b1a1	2	T2b	1	X2b+226	1
H1u	1	J1b1b1a	1	T2b19	1	X2c1a	1
H2a1	2	J1c1	1	T2b21	1		
H2a2b1	1	J1c2	2	T2b23	1		
H2a2b5a	1	J1c2b5	1	T2b27	1		

Haplogroups were identified using Phylotree[26] that was implemented in the MToolBox pipeline.

Supplementary Table 3. Homoplasmic variants in the 130 participants of LCL and whole blood sample.

Supplementary Table 4. Location and frequency of the 117 heteroplasmies of 581 in whole blood samples. Among the 581 sites being homoplasmy in blood samples, 117 (20.4%) sites were heteroplasmic in 62 blood samples.

Supplementary Table 5. Location and frequency of the 66 heteroplasmies of 608 in whole LCL samples. Among the 608 sites being homoplasmy in LCL samples, 66 (10.8%) were heteroplasmic in 41 LCL samples.

Supplementary Table 6. Comparison of heteroplasmic sites in their gene location and functional annotation between LCL and blood samples.

6a. Comparison of heteroplasmic sites in gene locations between LCL and WB

	A					B				
	LCL		WB			LCL		WB		
**Region**	**Count**	**%**	**Count**	**%**	***p***	**Count**	**%**	**Count**	**%**	***p***
D-loop	36	0.54	51	0.44	0.21	36	0.14	17	0.2	0.28
Coding	24	0.37	54	0.46	0.34	149	0.57	57	0.67	0.09
RNA	4	0.062	8	0.068	1	38	0.15	5	0.059	0.05
tRNA	2	0.030	2	0.017	1	30	0.12	5	0.058	0.17
Intergenic	0	0	1	0.0085	1	5	0.019	1	0.012	1
Total	66		117			258		85		

A. Heteroplasmic sites that are also homoplasmic sites: location and frequency of the 117 (of 581) heteroplasmic site in whole blood samples; location and frequency of the 66 (of 608) heteroplasmic sites in LCL samples. B. Heteroplasmic sites only: location and frequency of the 258 heteroplasmies in LCL samples; location and frequency of the 85 heteroplasmies in whole blood (WB) samples.

6b. Comparison of functional annotation between two types of heteroplasmic sites in LCL or WB samples

	A				B				P_{NS}	P_{CADD}
	Total n	Coding n	NS n (%)*	CADD≥15 n (%)#	Total n	Coding n	NS n (%)*	CADD≥15 n (%)#		
LCL	66	24	3 (13)	1 (4.2)	258	149	115 (77)	77 (51)	1.5e-9	4.0e-6
WB	117	54	15 (28)	4 (7.4)	85	57	29 (51)	20 (35)	0.028	0.0004

A. Heteroplasmic sites that are also homoplasmic sites: location and frequency of the 117 (of 581) heteroplasmic site in whole blood samples; location and frequency of the 66 (of 608) heteroplasmic sites in LCL samples. B. Heteroplasmic sites only: location and frequency of the 258 heteroplasmies in LCL samples; location and frequency of the 85 heteroplasmies in whole blood (WB) samples. CADD, combined annotation-dependent depletion (CADD) PHRED-like score ≥15. Nonsynonymous (NS) n(%)*, the number of nonsynonymous sites and the proportion of the sites among the total sites in protein coding genes; CADD≥ n(%)*, the number of deleterious sites and the proportion of the sites among nonsynonymous sites. P_{NS}, p-value for comparison of the proportion of nonsynonymous variants between two types of heteroplasmic sites (i.e., the sites with AAFs only in the 3%-97% range and the sites that being both heteroplasmic and homoplasmic) in coding variants. P_{CADD}, p-value for comparison of the proportion of deleterious variants between two types of heteroplasmic sites in coding variants.

Supplementary Table 7. Heteroplasmies across the 130 LCL-derived DNA samples

Supplementary Table 8. Heteroplasmies mutations across the 130 whole blood-derived DNA samples

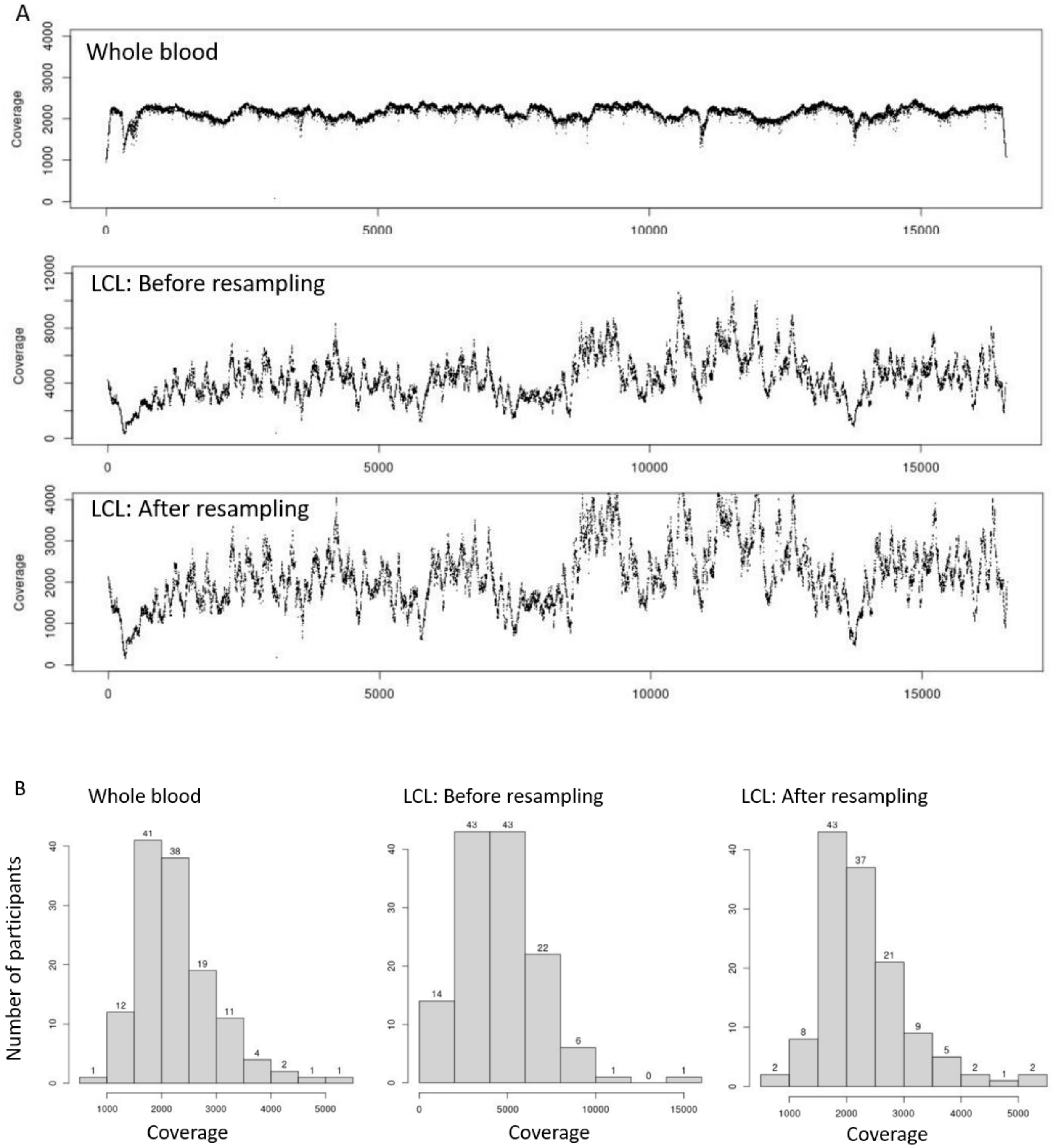

Supplementary Figure 1. **Sequencing coverage of the 130 whole blood samples and the paired 130 LCL samples of the same 130 Framingham Heart Study participants.** A. The distribution of median coverage of across mtDNA loci. B. The distribution of median coverage per sample. Before resampling, the median coverage in LCL samples was 4157-fold (inter quartile range 2846 to 5444).We performed resampling to harmonize sequencing coverage in LCL. We generated 10 packets with similar coverage distributions as that in whole blood samples (median 2177-fold (inter quartile range 1866 to 2578).

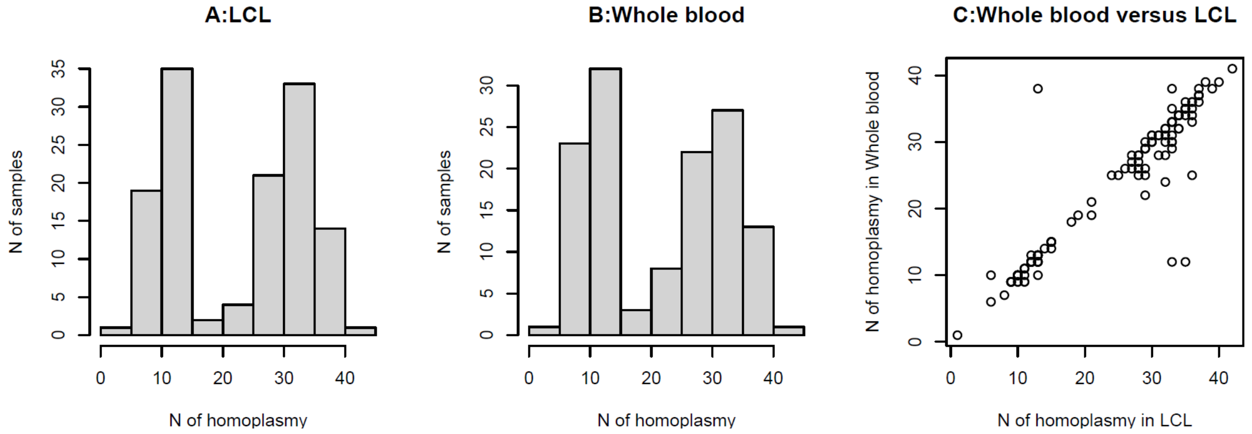

Supplementary Figure 2. The distribution of homoplasmic variants in 130 FHS participants. A. Histogram of number of homoplasmic variants carried by LCL samples. B. Histogram of number of homoplasmic variants carried by whole blood samples. C. Comparison of homoplasmic variants carried between paired LCL and blood samples. The median number of homoplasmic sites was 27 (interquartile range = 12, 32) per LCL sample and was 25 (interquartile range = 12, 33) per whole blood sample.

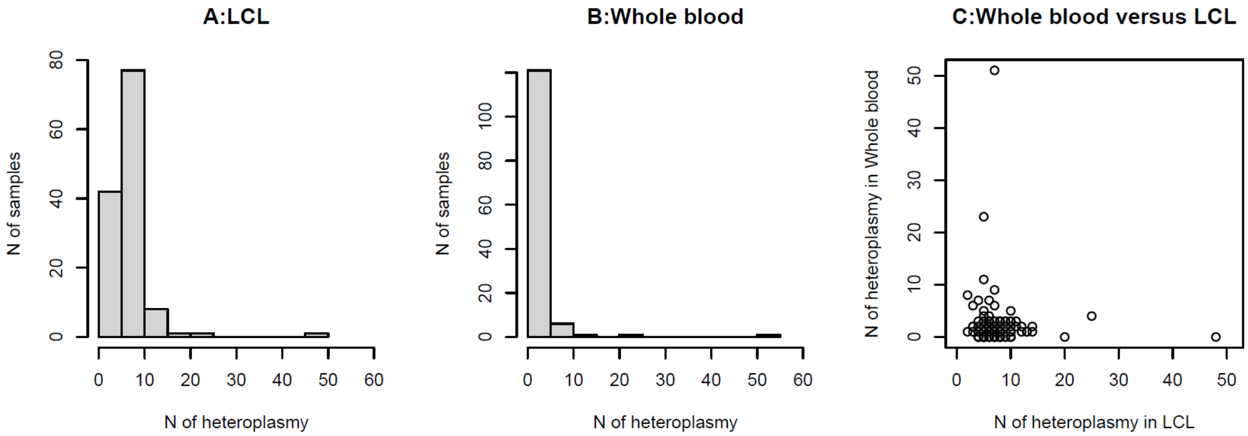

Supplementary Figure 3. Comparison of heteroplasmic mutations between paired samples. A. Histogram of number of heteroplasmic mutations carried by LCL-derived DNA samples. B. Histogram of number of heteroplasmic mutations carried by whole blood-derived DNA samples. C. Comparison of heteroplasmies carried between paired LCL and blood samples. At an individual level, a LCL sample carried a much higher number of heteroplasmic sites (median=7 with interquartile range = 5, 8) than a blood sample (median = 1 with interquartile range 0, 3).
